# A Systematic Review of the Impact of Physical Exercise-Induced Increased Resting Cerebral Blood Flow on Cognitive Functions

**DOI:** 10.3389/fnagi.2022.803332

**Published:** 2022-02-14

**Authors:** Maria B. Renke, Anna B. Marcinkowska, Sylwester Kujach, Paweł J. Winklewski

**Affiliations:** ^1^Functional Near Infrared Spectroscopy Lab, Department of Human Physiology, Medical University of Gdańsk, Gdańsk, Poland; ^2^Department of Electronics, Telecommunication and Informatics, Gdańsk University of Technology, Gdańsk, Poland; ^3^Applied Cognitive Neuroscience Lab, Department of Human Physiology, Medical University of Gdańsk, Gdańsk, Poland; ^4^Second Department of Radiology, Medical University of Gdańsk, Gdańsk, Poland; ^5^Department of Physiology, Gdańsk University of Physical Education and Sport, Gdańsk, Poland; ^6^Department of Human Physiology, Medical University of Gdańsk, Gdańsk, Poland

**Keywords:** aging, cerebral perfusion, cognition, dementia prevention, exercise

## Abstract

Brain perfusion declines with aging. Physical exercise represents a low-cost accessible form of intervention to increase cerebral blood flow; however, it remains unclear if exercise-induced amelioration of brain perfusion has any impact on cognition. We aimed to provide a state-of-the art review on this subject. A comprehensive search of the PubMed (MEDLINE) database was performed. On the basis of the inclusion and exclusion criteria, 14 studies were included in the analysis. Eleven of the studies conducted well-controlled exercise programs that lasted 12–19 weeks for 10–40 participants and two studies were conducted in much larger groups of subjects for more than 5 years, but the exercise loads were indirectly measured, and three of them were focused on acute exercise. Literature review does not show a direct link between exercise-induced augmentation of brain perfusion and better cognitive functioning. However, in none of the reviewed studies was such an association the primary study endpoint. Carefully designed clinical studies with focus on cognitive and perfusion variables are needed to provide a response to the question whether exercise-induced cerebral perfusion augmentation is of clinical importance.

## Introduction

Accelerated growth of the rate of cognitive impairment is an emerging problem of the senescent population. This might be explained due to the fact that the increased prevalence of cerebrovascular and neurodegenerative disorders correlates with age, which is one the greatest risk factors of late-onset Alzheimer's disease (AD) and other types of dementia (Hebert et al., 2010, 2013; Alzheimer's Association, 2019). The population aged over 65 years is constantly growing and is expected to be three times higher in 2050 than 2010, reaching 1.5 billion (WHO et al., 2011) and similarly, the number of patients suffering from AD (24 million cases in 2018) is estimated quadruple in 2050 (Dos Santos Picanco et al., 2016). These statistics may have gross social and economic implications, considering that the annual cost of care per patient with dementia is on average 3.5 times higher than for a person without any type of dementia (Hebert et al., 2013; Alzheimer's Association, 2018).

### Cerebral Blood Flow

Cerebral blood flow (CBF) is one of the most commonly used parameters of brain function. It is defined as the rate of blood delivered by the arteries to the capillary bed in brain tissue and is calculated as the volume of blood in milliliters per 100 g of the cerebral tissue per minute (Bertsch et al., 2009). The local neuronal metabolism and activity are strongly correlated with CBF and this relationship is referred to as neurovascular coupling (Kisler et al., 2017). The rate of CBF is known to be diminished in several neuropsychiatric conditions, for example, mild cognitive impairment (MCI), Alzheimer's disease, vascular dementia and Huntington's disease (Ferris et al., 1980; Berent et al., 1988; Stirling Meyer et al., 1988; Alexander et al., 1997). Several studies exist that have shown the correlation between the rate of CBF and the clinical state of the patient, specifically, the cognitive functioning level. Monitoring CBF is often simpler and more reliable in clinical conditions than assessing cognitive functions, especially in older individuals (Lacalle-Aurioles et al., 2014). Various imaging techniques, such as positron emission tomography (PET), magnetic resonance imaging (MRI) with contrast (DSC-MRI), arterial spin labeling MRI (ASL-MRI), and Doppler ultrasonography, are used to assess the rate of CBF. Assessment of neuronal activity is moreover allowed through the blood oxygen level-dependent (BOLD-MRI) signal and can also be achieved by near-infrared spectroscopy (NIRS).

### CBF and Aging

In healthy aging, the decrement in mental abilities is not primarily caused by hypoperfusion; on the contrary, the level of CBF is more than sufficient compared to the demand of neurons (Stefanovic et al., 2004; Pasley et al., 2007; Hutchison et al., 2013; Nealon et al., 2017). The rate of CBF decreases constantly, typically 0.35–0.45% per year, for subjects who are middle-aged and older (Leenders et al., 1990; Parkes et al., 2004). Simultaneously, the risk of developing mild cognitive impairment grows due to gradual loss in cognitive functions (Wolters et al., 2017). The reason for the decline of CBF associated with aging is not entirely clear; however, it could be explained by the changes in the density and elasticity of cerebral blood vessels, and neuronal degeneration, as well as the reduced activity of pericytes (Kalaria, 1996; Zhang et al., 2017).

### Physical Activity, Cerebral Blood Flow, and Cognitive Functions

Chronic physical exercise is believed to have a multifactorial impact on cerebral function (Barnes, 2015), including the elevation of perfusion levels (Gligoroska and Manchevska, 2012). Moreover, physical activity is a low-cost accessible form of intervention. These effects could potentially be applied in dementia and cognitive decline prevention programs (Daviglus et al., 2010). The effect of exercise on resting CBF, and consequently, on cognitive functions, has been tested in various groups of patients, however, such findings have not yet been systematically reviewed.

It has been shown that physical exercise modulates CBF (Querido and Sheel, 2007; Smith and Ainslie, 2017). Acute aerobic, mild-to-moderate intensity exercise could increase CBF, whereas high-intensity exercise (above anaerobic threshold) leads to CBF decline (Smith and Ainslie, 2017). Moreover, a study evaluating acute resistant/strength exercise effects has demonstrated a reduction in CBF (Perry and Lucas, 2021). With regard to chronic exercise, the studies conducted thus far suggest an improvement in CBF as well as cerebrovascular reactivity (Ainslie et al., 2008; Murrell et al., 2013). These beneficial changes may result from the post-physical training brain vascularization development that is associated with brain metabolic changes, as well as changes in the blood vessels themselves (Ainslie et al., 2008; Steventon et al., 2018). Therefore, type, intensity, time and duration of physical exercise could evoke different responses in CBF (Ainslie et al., 2008; Smith and Ainslie, 2017; Perry and Lucas, 2021).

A lower rate of resting CBF is known to correlate with a worsened neuropsychological outcome, whereas the commonly observed amelioration of cognitive functions (executive functions, working memory) are usually explained by post-exercise synthesis/release of brain-derived neurotrophic factor (BDNF) concentration and central catecholamine synthesis (Chang et al., 2012; Mcmorris and Hale, 2012). Whether a permanent increase of CBF attained by regular training is typically followed by ameliorated cognitive functioning has not yet been well proved. In this systematic review, we aimed to provide a state-of-the-art summary of the current knowledge regarding the relationship between physical exercise, resting CBF and cognition.

## Materials and Methods

The literature analyzed in this article was selected by a comprehensive search of the PubMed (MEDLINE) database. All the trials published up until the 17th of July 2021 were taken into account. Pubmed (MEDLINE) database was chosen because it represents an unfiltered source of primary literature comprising all different kinds of publication types occurring in academic journals.

To include all the research on the effects of regular physical activity on CBF and cognitive functions, the following search term was composed of the relevant key words (“fitness” or “exercise” or “physical activity” or “training”) and (“cerebral blood flow” or “CBF” or “MRI” or “ASL” or “PET” or “NIRS” or “SPECT” or “Doppler” or “BOLD”) and (“cognitive function” or “cognitive testing” or “cognition”).

The results were filtered to show only clinical trials and then manually searched and further qualified for the study by 2 independent blinded reviewers.

A two-step approach was used to select articles: (1) titles and abstracts of all search results were screened for the following characteristics (a) original article published in English, (b) case studies and children studies were excluded; (2) full-text articles were obtained from the selected studies and were reviewed on the following inclusion criteria: (a) physical exercise intervention, (b) measurement of the CBF rate, (c) cognitive functioning assessment. All the included studies matched the focus of our review, which included an investigation of the effects of a long-term physical exercise intervention on adults, measurement of perfusion after the training period and preferably, before the training period, and the assessment of cognitive function and its referral to CBF.

## Results

As a result of the search phrase, 125 results were shown in the PubMed database; however, several papers were excluded for the following reasons: (1) no physical exercise intervention in the study or physical exercise was solely a means to achieve some physiological state, for example, dehydration (*n* = 56), (2) 19 of the search results were study protocols, which shows that this area might be planned for future investigation, (3) no measurement of the CBF rate (*n* = 21), (4) no cognitive testing (*n* = 2), and (5) the cognitive results were not conducted after the physical exercise or did not match the area of CBF examination because those measurements were not the main focus of the study (6) participants under the age of 18 (*n* = 2) (Davis et al., 2011; Cho et al., 2017). Four of the studies combined physical exercise with cognitive training, indicating a possible next step in the interventions of cognitive impairment, although they cannot be easily compared with the studies included.

**Figure 1:**
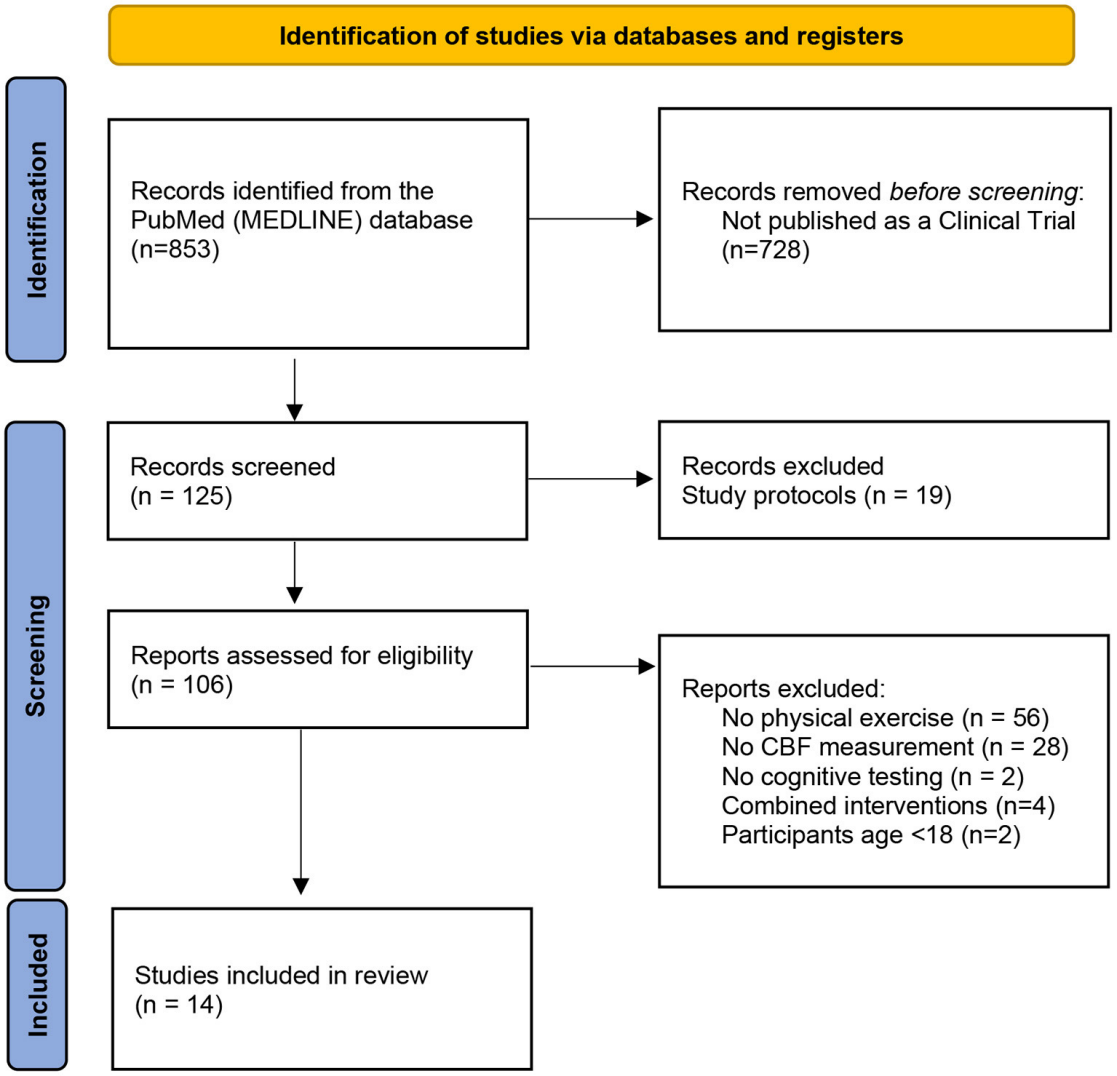
PRISMA (Preferred Reporting Items for Systematic Reviews and Meta-Analyses) flow chart showing literature selection. From Page et al. (2021).

A total of 14 studies matched the search criteria and the three main points of the focus of our analysis (Figure 1). All the articles analyzed described longitudinal clinical trials. Nine of the studies (Stanek et al., 2011; Moore et al., 2015; Castellano et al., 2017; Shimizu et al., 2018; Stringuetta Belik et al., 2018; Cho and Roh, 2019; Northey et al., 2019; Guadagni et al., 2020; Lehmann et al., 2020) conducted well-controlled exercise programs that lasted 12–19 weeks for 10–40 participants and two studies (Rosano et al., 2010; Espeland et al., 2018) were conducted in much larger groups of subjects for more than 5 years; however, the amount of exercise was indirectly measured, and three studies (Decroix et al., 2016; Lefferts et al., 2016; Olivo et al., 2021) were focused on acute exercise. Three were pilot studies. The experiments were conducted on various patient samples, including older women, patients undergoing haemodialysis and cardiac rehabilitation, patients with diabetes mellitus, female breast cancer survivors, patients recovering after stroke, and patients suffering from mild AD and MCI.

The rate of perfusion was measured using transcranial Doppler ultrasound examination (*n* = 6) (Stanek et al., 2011; Lefferts et al., 2016; Stringuetta Belik et al., 2018; Cho and Roh, 2019; Northey et al., 2019; Guadagni et al., 2020), functional NIRS (fNIRS) (*n* = 2) (Decroix et al., 2016; Shimizu et al., 2018) and MRI (*n* = 5) (Rosano et al., 2010; Moore et al., 2015; Espeland et al., 2018; Lehmann et al., 2020; Olivo et al., 2021), including both ASL and BOLD studies. BOLD studies were included as they represent changes of deoxyhaemoglobin concentration, which are related to blood supply mechanisms (Wang et al., 2021). In addition, one PET study (Castellano et al., 2017) was included, as brain glucose metabolism closely follows perfusion rate (Baron et al., 1982).

Cognitive functions were assessed by suitable tests that could be compared with the perfusion measurement results. Because of the variety of the used tests, they will be described in detail in the paragraphs dedicated to specific studies.

A summary of the intervention study designs is provided in Tables 1–3.

**Table 1:** Summary of long-term lifestyle interventions.

**References**	**Sample description and size (mean age)**	**Description of the method**	**Duration of intervention**	**Aim for weekly activity**	**CBF measurement**	**CF measurement**	**Statistically significant imaging results**	**Statistically significant cognitive results**
Espeland et al. (2018)	Patients diagnosed with diabetes 157 EXP (57.5 ± 6.3) 153 CON (58.5 ± 6.6)	Intensive lifestyle intervention	10–12 years	>175 min/week	MRI	Rey auditory verbal learning test, digit symbol coding Test, SCWT, MMSE	CBF level (*p* < 0.05)	Digit symbol coding, SCWT, TMT part B (*p* < 0.05)
Rosano et al. (2010)	Older patients, PA—active and after PE intervention, SA—not active after no PE intervention 20 EXP (81.45); 10 CON (80.8)	Walking, follow-up 2 years after intervention, after maintaining active/not active lifestyle	26+ weeks	150 min/week	fMRI while DSST	fDSST, MMSE	Activation was higher in EXP group, *p* = 0.04	fDSST: more widely distributed network that included ECF regions within the dorsolateral prefrontal, posterior parietal, and anterior cingulate cortices compared with the regions active in the CON group

*CBF, cerebral blood flow; EXP, experimental group; CON, control group; MRI, magnetic resonance imaging; SCWT, Stroop Color and Word Test; MMSE, Mini-Mental State Examination; TMT, Trial Making Test; PE, physical exercise; CF, Cognitive Function; fMRI, functional magnetic resonance imaging; DSST, Digit Symbol Substitution Test*.

**Table 2:** Summary of controlled exercise program studies.

**References**	**Sample description and size (mean age)**	**Description of the method: type of intervention, duration of intervention, duration of session, frequency**	**CBF measurement**	**CF measurement**	**Statistically significant imaging results**	**Statistically significant cognitive results**
Cho and Roh (2019)	Older women 19 EXP (68.9); 18 CON (69)	Taekwondo 16 weeks; 60 min 5 × /week	Transcranial Doppler ultrasonography	MMSE; SCWT	No *p* < 0.05 results	SCWT test (*p* = 0.022)
Stringuetta Belik et al. (2018)	Haemodialysis patients 15 EXP (50.3); 15 CON (57.8)	Aerobic training, cycle ergometer Four months; 30 min increasing toward 45 min; 3 × /week	Transcranial Doppler ultrasonography	MMSE	Maximum cerebral arterial flow velocity per area (*p* = 0.002)	MMSE (*p* = 0.023)
Northey et al. (2019)	Female cancer survivors 17 (HIIT *n* = 6, MOD *n* = 5, CON *n* = 6) (62.9)	HIIT or moderate training 12 weeks; 20–30 min; 3 × /week	Transcranial Doppler ultrasonography	CogState battery, verbal learning, episodic memory, executive function, test working memory	No *p* < 0.05 results; a large-sized effect for HIIT in comparison to CON for resting MCAvmean	Episodic memory (moderate effects for both HIIT and MOD); executive function and working memory (HIIT group had large effects) in comparison to CON, between groups no significant differences
Moore et al. (2015)	Patients after stroke 20 EXP (68); 20 CON (70)	Community exercise vs. stretching 19 weeks; 45–60 min; 3 × /week	MRI	ACE-R	Elevated CBF in medial temporal lobe region	ACE-R (*p* < 0.01)
Shimizu et al. (2018)	Older adults 30 EXP; 9 CON 74.90 MMT, 73.33 STT	Movement music therapy and single training task 12 weeks; 60 min in total; 1 × /week	fNIRS	FAB test	Significantly higher activation between groups in Brodmann Area 10	Significant improvement in FAB after MMT, no after STT; however, no significant difference between the groups
Castellano et al. (2017)	Patients with mild AD 10 EXP (73)	Walking on a treadmill 12 weeks; Ph1 6 weeks 15–40 min (adding 5 min weekly); Ph2 40 min; 3 × /week	PET-CT	3MS; HVLT; Verbal Digit Span; SCWT; DSST	Significantly higher global glucose metabolism	Shorter completion time on the TMT Awas related to higher global CMRacac (*p* = 0.01); A tendency toward improvement on condition 2 (color naming) of the SCWT
Stanek et al. (2011)	Cardiac rehabilitation patients 51 EXP (67.75) [36 stress test subset (68.72), 42 CBF measurement (68.17)]	Phase II CR program up to 12 weeks; 60 min; 3 × /week	Transcranial Doppler ultrasonography	3MS; Attention-Executive-Psychomotor Trail Making Test A and B; Grooved Pegboard-dominant hand; FAB; Letter-Number Sequencing subtest of Wechsler Adult Intelligence Scale-III, Language- Boston Naming Test -Short form; Animal Naming	Significant change in ACA flow velocity (*p* = 0.03) in a subset of 42 individuals	Significant improvements: 3MS; Attention-Executive-Psychomotor (Letter-Number sequencing, Grooved Pegboard); HVLT (learning trial recall trial); Brief visuospatial memory test (learning trial, recall trial)
Guadagni et al. (2020)	Healthy low-active middle-aged and older adults 206 EXP (69.5 ± 6.4 years old)	Controlled exercise program aerobic exercise 6 months; increased 20–40 min; 3 × /week	Transcranial Doppler ultrasonography	Card Sorting Test, SCWT, SDMT, Buschke Selective Reminding Test, Medical College of Georgia Complex Figure, Verbal Fluency Test from the Delis-Kaplan Executive Function system, Auditory Consonant Trigram Test	Resting baseline VP (*p* = 0.014); resting baseline CVCi (*p* = 0.005) determinations increased. A decrease in resting baseline CVRi was also found (t202 = 3.378, *p* = 0.001)	Positive changes before and after intervention in the executive functions/processing speed (*p* = 0.029), executive functions/concept formation (*p* = 0.02), verbal memory (*p* = 0.001), and fluency (*p* = 0.004) domains were observed. The figural memory domain showed a negative change (*p* < 0.001)
Lehmann et al. (2020)	34 healthy, right-handed adults of either sex 15 EXP (23); 16 CON (23.5)	Controlled exercise program cardiovascular exercise 2 weeks; 19 min; 3–4/week	fMRI	Motor learning time	Significant between group difference in frontal brain areas	Group EXELEARN learned the DBT at a significantly higher rate compared with RESTLEARN, *p* = 0.025

*EXP, experimental group; CON, control group; MMSE, Mini-Mental State Examination; SCWT, Stroop Color and Word Test; MCAvmean, mean blood velocity in middle cerebral artery; ACE-R, Addenbrooke's Cognitive Examination Revised; fNIRS, functional near-infrared spectroscopy; FAB, Frontal Assessment Battery; AD, Alzheimer's disease; DSST, digit symbol substitution test; CMRacac, cerebral metabolic rate of acetoacetone; ACA, anterior cerebral artery; MMT, music movement therapy; STT, standard training therapy; HIIT, high-intensity interval training; MOD, moderate intensity exercise group; CR, Cardiac Rehabilitation; PET-CT, positron emission tomography; DBT, dynamic balancing; TMT, Trial Making Test; HVLT, Hopkins Verbal Learning Test; fMRI, functional magnetic resonance imaging; 3MS, Modified Mini-Mental State*.

**Table 3:** Summary of the acute exercise studies.

**References**	**Sample description and size (mean age)**	**Description of the method, duration of exercise**	**CBF measurement**	**CF measurement**	**Statistically significant imaging results**	**Statistically significant cognitive results**
Lefferts et al. (2016)	University community 15 men (22) 15 women (20 ± 3)	Cycle ergometer (hypoxic and normoxic exercise compared), 20 min	Transcranial Doppler ultrasonography	Eriksen Flanker test (executive function); N-back number task (working memory)	No significant differences	Differences in caution between normoxia and hypoxia
Decroix et al. (2016)	Healthy, well-trained men, some received coconut flavanoa oil 12 (30 ± 3)	Cycle ergometer, 30 min	fNIRS	SCWT	Significantly increased d(HBO_2_, HHB, HBtot)	Increased speed of information processing (RT)
Olivo et al. (2021)	Older adults 49 24 EXP (69.6 ± 2.8) 25 CON (70.7 ± 3.1)	Cycle ergometer, 30 min	ASL	N-back task, MMSE	Elevated CBF between groups	No significant results between groups

*fNIRS, functional near-infrared spectroscopy; SCWT, Stroop Color and Word Test; ASL, arterial spin labeling; MMSE, Mini-Mental State Examination; HbO_2_, oxygenated hemoglobin; HHb, deoxygenated hemoglobin; HBtot, total hemoglobin; CF, Cognitive function; RT, reaction time*.

### Long-Term Lifestyle Interventions

#### Intensive Lifestyle Intervention and Cerebral Blood Flow in Diabetes Mellitus Patients

Diabetes mellitus may impair CBF through mechanisms that include vessel stiffness, poor vascular function and lumen narrowing (Nealon et al., 2017). In order to investigate the possibility of slowing these processes down, an intensive lifestyle intervention (ILI) was performed in a long-term study by Espeland et al. (2018) as a part of the Look AHEAD (Action for Health in Diabetes) multicentre, randomized controlled clinical trial (Ryan et al., 2003). Patients diagnosed with diabetes were randomly assigned into two groups. The ILI group (*n* = 157) underwent a process that included diet modification and physical activity (goal was more than 175 min of activity per week) to induce and maintain an average weight loss ≥7%. During the first 6 months, the participants were evaluated weekly and then three times per week for the following 6 months. In the following years, actions were taken in order to encourage the maintenance of the lifestyle change in the patients. The participants in the second group (*n* = 153) received a control condition of diabetes support and education (DSE), which included three group sessions each year, however, no specified diet, activity or weight goals. CBF was measured using ASL MRI at the 10–12-year anniversary of Look AHEAD enrolment for each subject. Cognitive testing was focused on verbal learning and memory (Rey Auditory Verbal Learning Test), processing speed and working memory [Digit Symbol Substitution Test (DSST)], executive functions [Modified Stroop Color and Word Test (SCWT) and the Trail-Making Test Part B (TMT B)], and global cognitive function [Modified Mini-Mental State Examination (MMSE)]. The ASL-MRI results showed a significant improvement in CBF level in the ILI group compared to the DSE group (*p* = 0.04) and the level of CBF was 6–7% higher in all regions for the ILI participants, compared to DSE, with minor interregional differences between the groups (*p* = 0.95). A significant difference was also observed (*p* < 0.05) in three of the cognitive assessment results (DSST, SCWT, and TMT B). Neuropsychological results indicate better performance in psychomotor speed after ILI, while memory and executive functioning did not show improvement. Higher CBF levels were associated with poorer composite cognitive scores in the DSE participants, but not in the ILI group. The possible explanation is that this reflects an adaptive response to greater metabolic requirements related to poorer cognitive efficiency through vascular dilation or angiogenesis (Loane and Kumar, 2016; Daulatzai, 2017). In the ILI group, no overall association between CBF and cognitive functioning was found. The authors of the study proposed an explanation linked to a blunted neurovascular response to decreases in cognition and neurodegeneration, including weight loss-induced alterations in apelin and leptin levels (Castan-Laurell et al., 2008; Abbenhardt et al., 2013), which are hormones that may promote angiogenesis and vasodilatation (Busch et al., 2011; Castan-Laurell et al., 2011; Khazaei and Tahergorabi, 2015; Sawicka et al., 2016) or decreases in cardiac output, which may lead to lower CBF independent of blood pressure (Lingzhong et al., 2015).

#### Psychomotor Speed and Functional Brain MRI 2 Years After Completing a Physical Activity Treatment

In the pilot study conducted by Rosano et al. (2010), 30 elderly participants took part in a follow-up 2 years after a 1-year treatment that consisted of BOLD-MRI examination, MMSE and DSST. Twenty of the participants completed a physical activity lifestyle intervention and remained active afterwards, and 10 patients who were in the control group (“Successful Aging” group) maintained <20 min a week of regular exercise. The imaging results showed a statistical difference in overall activation levels. Simultaneously, the results of DSST indicated a more widely distributed network that included executive function regions in the experimental group within the dorsolateral prefrontal, posterior parietal, and anterior cingulate cortices, compared with the active regions in the control group.

### Controlled Exercise Program Studies

#### Aerobic Training in Different Age Groups of Healthy Subjects

In a study conducted by Cho and Roh (2019) researched the effects of Taekwondo exercise interventions on CBF and the cognitive functions of elderly women.

Participants were randomly assigned to Taekwondo or control groups, the intervention lasted 16 weeks and 60-min sessions were attended by the participants five times per week. CBF was measured using Doppler ultrasonography and the cognitive testing was tailored to the age of participants.

In the group of elderly women, the purpose of physical exercise is to delay the loss of cognitive function associated with aging (Cho and Roh, 2019). The study sample consisted of the taekwondo group (*n* = 19) with a mean age of 68.89 years and control group (*n* = 18), mean age 69 years. The findings regarding the cerebral blood velocity measurements were not statistically significant; however, cognitive functions were significantly ameliorated in the taekwondo group. Specifically, the SCWT score exhibited a significant difference (*p* = 0.022) between the groups, which indicates improvement in executive functions. Serum levels of neurotrophic grow factors (BDNF, VEGF, and IGF-1) were also significantly higher in the taekwondo group.

The study performed by Guadagni et al. (2020), analyzed a single group of 206 middle-aged and older participants (aged 69.5 ± 6.4 years), comparing results collected before and after a 6-month aerobic exercise intervention. Both assessments consisted of a transcranial Doppler ultrasonographic examination and a cognitive test battery, including the Card Sorting Test, SCWT, DSST, Buschke Selective Reminding Test, Medical College of Georgia Complex Figure, Verbal Fluency Test from the Delis-Kaplan Executive Function system, or the Auditory Consonant Trigram Test. Pre- and post-intervention differences in the physiological examination were found in baseline mean peak blood flow velocity and baseline cerebrovascular conductance index (an increase and a decrease of cerebrovascular resistance index). Cognitive testing revealed multiple positive changes before and after intervention in the executive functions/processing speed (*p* = 0.029), executive functions/concept formation (*p* = 0.02), verbal memory (*p* = 0.001), and fluency (*p* = 0.004) domains. The figural memory domain showed a negative change (*p* < 0.001).

A cohort of 18–35 years old participants was examined by Lehmann et al. (2020), with an experimental group of 15 subjects that completed a 2-week cardiovascular exercise program. There was a significant difference in BOLD-MRI results with higher activity level in the frontal brain areas, compared to a 16-participant control group. Additionally, motor learning time proved to be significantly shorter in the experimental group with *p* = 0.025.

#### Aerobic Training and Mild AD

Alzheimer's Disease is usually associated with a substantial decline in the rate of CBF, up to 40 percent compared to healthy individuals (De la Rosa et al., 2020). This decrease is most prominent in regions such as the precuneus, the hippocampus, the posterior cingulate gyrus and the temporal, occipital and parietal lobes (Johnson et al., 2005; Du et al., 2006; Asllani et al., 2008; Austin et al., 2011; Binnewijzend et al., 2013). Regular physical activity is known to increase the lowered level of CBF in areas such as the hippocampus and anterior cingulate gyrus in older adults (Ainslie et al., 2008; Burdette et al., 2010; Heo et al., 2010).

In the study conducted by Castellano et al. (2017) a group of 10 participants, average 73 years old, underwent a 3-month aerobic training program, consisting of 3 sessions a week of walking on the treadmill. The intervention was divided into two 6-week phases, in the first phase, the length of the sessions was increasing by 5 min weekly, finishing at 40 min, which was maintained until the end of the intervention.

The significantly elevated global glucose metabolism, which is an indicator of the elevated global perfusion rate (Baron et al., 1982), was observed on PET imaging after the intervention. Cognitive results were not significantly elevated; however, observed was a tendency toward improvement in executive functions [better results incondition 2 (color naming) of the SCWT (*p* = 0.06)].

Due to the relatively small group of participants in the study, the results should be treated as preliminary.

#### Physical Exercise in Mild Cognitive Impairment

A study conducted by Shimizu et al. (2018) was designed to compare the effect of a regular physical activity workout program and a program using the same movements, but with percussion music accompaniment (movement music training). All the participants were diagnosed with MCI. The group of patients taking part in the standard training therapy program (STT group) consisted of nine participants with mean age of 73.33 years and the music movement therapy (MMT group) consisted of 30 participants, mean age 74.9 years. CBF was assessed by fNIRS before and after the 12-week intervention and cognitive functions were measured on the same days, using frontal assessment battery (FAB). During the 3 months of the training program, the participants met once a week for a 60-min session. Post-intervention results showed a significantly higher activation between MMT and STT in Brodmann Area 10 of the prefrontal cortex and significant improvement in FAB results following MMT, compared to post-intervention outcomes of the STT; however, a *p*-value = 0.088 implicates some possible improvement of the results. No significant difference in cognitive results was observed between the groups.

The much smaller STT group is a big limitation of this study, as it is over three times smaller than the MMT group, mainly questioning the observed difference in significance of cognitive function measurement, and furthermore, no control group that would not undergo any exercise program.

#### Cardiac Rehabilitation and Cognitive Function

Patients suffering from cardiovascular disease (CVD) often experience an accelerated loss of cognitive abilities (Moser et al., 1999; Singh-Manoux et al., 2003) for multiple reasons, including low cardiac output (Jefferson et al., 2007), endothelial function (Moser et al., 2004), poor cardiovascular fitness (Gunstad et al., 2005) and CBF (Dai et al., 2008). Stanek et al. (2011) conducted a study to determine the effects of cardiac rehabilitation (CR) on the brain on a group of 51 patients with a mean age 67.75 years.

The patients were enrolled in a CR program (phase II CR program at Summa Health System's Akron Hospital, Ohio) customized to each individual with a duration of up to 12 weeks, three meetings per week, with 40 min of aerobic exercise for each session. Neuropsychological testing was focused on deriving a complex picture of each patient's cognitive functioning. Significant improvement was found upon analysis of the modified MMSE results (*p* = 0.04), Attention-Executive-Psychomotor function (observed through a significant improvement in Letter-Number Sequencing test, *p* = 0.02) and Grooved Pegboard-dominant hand test (*p* = 0.02). Moreover, a significant improvement (*p* < 0.001) was found in all the performed memory tests (Hopkins Verbal Learning Test—Revised: learning and delayed recall, Brief Visual Memory Test—Revised: learning, and delayed recall) from baseline to 12-week follow-up. CBF was evaluated in a subset of 42 individuals using transcranial Doppler ultrasonography and a significant improvement in anterior cerebral artery flow velocity was noted.

#### Community Exercise Therapy Following Stroke

Stroke and reduced levels of CBF are among many cerebrovascular dysfunctions induced by common metabolic abnormalities, including impaired glucose control, dyslipidemia, hypertension, obesity and low cardiorespiratory fitness (Caplan and Hennerici, 1998; Creager et al., 2003; Versari et al., 2009; Kernan et al., 2014). One of the main reasons for an accelerated process of age-related decline in CBF, brain atrophy and cognitive functions following stroke is the patient's low level of physical activity in particular (Rand et al., 2009). The community exercise therapy described in the study design of Moore et al. (2015) aimed to investigate post-stroke functional benefits in patients. A total of 40 participants were randomly divided into two groups, an exercise group (*n* = 20, mean age = 68 ± 8 years) and a control group (*n* = 20, mean age = 70 ± 11 years); 37 participants experienced an ischemic stroke, and three, a haemorrhagic stroke. The exercise group completed a 19-week (three times a week, 45–60 min each session) exercise program that consisted of strength and balance exercises of increasing intensity, measured by heart rate monitors. The initial goal was 40–50% maximum heart rate at first, progressing toward 70–80%, with a 10% increase every 4 weeks, and the control group completed a matched-duration home stretching program. ASL-MRI imaging was performed both pre-intervention and post-intervention and no change in the global gray matter CBF post-intervention was observed. Regional blood flow of the medial temporal lobe in the exercise group was significantly increased (*p* = 0.05), but a between-group difference was not observed. Overall cognition, measured by Addenbrooke's Cognitive Examination Revised, improved with exercise (*p* < 0.01), which confirmed the hypothesis of this study.

#### Intradialytic Aerobic Training

Chronic kidney disease (CKD) is a risk factor for cognitive impairment (Kurella Tamura et al., 2008, 2011; Yaffe et al., 2010; Etgen et al., 2012) and up to 60% haemodialysed patients experience this condition (Murray et al., 2006; Kurella Tamura et al., 2011). One of the possible mechanisms responsible for the accelerated decrease of cognitive functioning is CVD among CKD patients. Stringuetta Belik et al. (2018) investigated the effects of intradialytic aerobic training on CBF (measured by transcranial Doppler ultrasound) and cognitive functions (with MMSE) in haemodialysis patients. The study participants (*n* = 30) were randomly assigned into two groups of 15. The intervention group (*n* = 15, mean age = 50.3 ± 17.24 years) participated in a 16-week exercise program, three times a week of sessions of aerobic activity on a cycle ergometer, gradually increased in duration (starting with 30 min and finishing at 45 min), and the control group of the equal size (mean age = 57.8 ± 15.01 years) maintained a regular lifestyle. Analysis of the post-intervention data found a significant difference between the groups in the transcranial Doppler examination results, the maximum cerebral arterial flow velocity (MCA-V) per area (*p* = 0.002), mean cerebral arterial flow velocity per area (*p* = 0.038), as well as the pulsatility index (*p* = 0.015) and a significant difference in the MMSE results (*p* = 0.023).

#### High-Intensity Interval Exercise and Moderate-Intensity Exercise in Breast Cancer Survivors

Seventy-five per cent of cancer survivors report cognitive impairment during and after treatment, particularly in the domains of working memory, executive functions and memory performance (Janelsins et al., 2014; Pendergrass et al., 2018).

The possible mechanisms by which cancer treatment may impact cognition are similar to age-related effects on the brain, although the decline tends to be more rapid in cancer survivors (Janelsins et al., 2014; Wefel et al., 2015; Ehlers et al., 2016; Zimmer et al., 2016). In a pilot study, performed by Northey et al. (2019), 17 women over the age of 50 years (mean age, 62.9 years) in remission of breast cancer were randomly allocated into three study groups. There were two exercise groups completing a 12-week exercise program (three times a week, 20–30 min of exercise), high-intensity interval training (HIIT) group (*n* = 6) and moderate exercise group (*n* = 5), as well as a control group (*n* = 6). CBF, measured by transcranial Doppler, showed no statistical difference in mean blood flow velocity of the middle cerebral artery (MCA_vmean_) between the HIIT and moderate exercise groups. There was a large-sized effect (*d* = 0.86) for the HIIT in comparison to the CON for resting MCA_vmean_. The outcomes of the cognitive function assessment, measured using tasks from the CogState battery, showed no statistically significant group × time interaction effects for verbal learning, episodic memory, executive function or working memory. However, both the HIIT and moderate-intensity exercise groups had moderate-sized effects for episodic memory in comparison to the control group. Although examined groups were relatively small (5-6 subjects).

### Acute Exercise Studies

Two studies, conducted by Decroix et al. (2016) and Lefferts et al. (2016) examined the effect of acute exercise, 20 min and 30 min sessions, on a cycle ergometer in a group of young adults (respectively, mean age 22.2 and 30 years).

The first study compared the effects of exercise during normoxia and hypoxia in a group of 30 participants. The authors found no significant differences in transcranial Doppler ultrasonography measurements and a statistical difference in caution, measured by a battery of cognitive tests, consisting of the Eriksen Flanker test and N-back number task.

In the second study, which included 12 participants, the effect of exercise was individually examined and paired with cocoa flavanol oil supplementation. In the fNIRS imaging, d(HBO_2_, HHB, HBtot) were increased after exercise. Stroop Color and Word test results showed significantly increased speed of information processing post-exercise.

A group of 49 older adults (mean age, 69.6 ± 2.8 years in the experimental group of 24 participants and 70.7 ± 3.1 years in the control group) similarly completed a 30 min cycling session. Subjects were afterwards examined with ASL-MRI, revealing a significant elevation in CBF levels in the exercise groups and with N-back task and MMSE. No significant difference was found in the cognitive results between the groups.

## Discussion

In this review, the studies of the relationships between physical exercise, CBF and cognitive functions were summarized.

From a theoretical point of view, there are three potential scenarios associated with aging-related cognitive deterioration: (1) decline in neuron number and function, (2) diminished cerebral perfusion, or (3) a combination of both factors. From a clinical perspective, simultaneous neuronal and perfusion decline is most likely a typical scenario.

For instance, in neurodegenerative disorders, the decrease in CBF could be implicated by the lowered energy demand of neuronal cells. Physical activity can stimulate these cells through multiple mechanisms of noradrenergic activation, elevation of lactic acid levels in blood, and increasing the release of BDNF (Lu et al., 2015), among other mechanisms. In these conditions, a CBF increase might not be directly correlated with effect on cognitive functions due to relative blood oversupply (compared to metabolic demand).

Such mechanism might have played a role in described studies in AD (Castellano et al., 2017; Shimizu et al., 2018) where increase in glucose metabolism (Castellano et al., 2017) and activation measured with BOLD signal (Shimizu et al., 2018) were not associated with any changes in cognitive functioning. AD is typically associated with glucose hypometabolism (Guan et al., 2021; Librizzi et al., 2021). Thus, reversal of glucose metabolism decline should be seen as a positive outcome. Significance of the reported findings (amelioration in metabolism/regional perfusion with no cognitive improvement) is nevertheless limited by small numbers of investigated patients.

**Figure 2:**
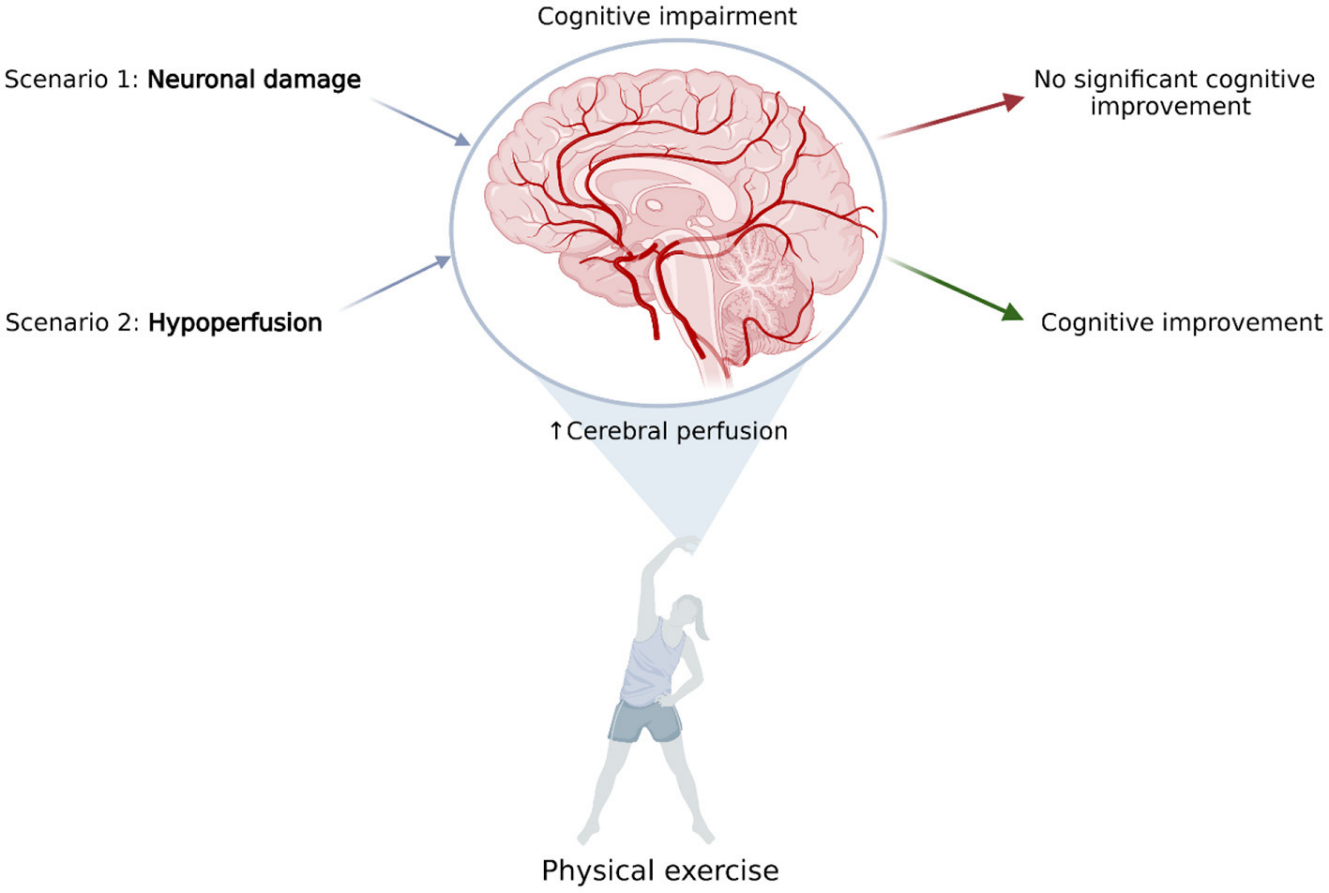
The effect of elevated cerebral blood flow, resulting from physical exercise on common scenarios of cognitive impairment. Scenario 1: Cognitive impairment is caused by neuronal damage; for example, in neurodegenerative diseases. Elevating cerebral perfusion is not efficient due to relative blood oversupply. Scenario 2: Cognitive impairment is caused by hypoperfusion; for example, in vascular disease. Elevating cerebral blood flow may improve patients' cognitive abilities. Proposed mechanisms have yet to be proved experimentally (Created with BioRender.com).

In contrast, cognitive impairment, correlated with permanent hypoperfusion, has been described in vascular diseases. Thus, cardiovascular improvement, induced by regular physical exercise (for example, cardiac rehabilitation), should have a positive effect on cognitive functions because it would target the limiting factor of diminished CBF (Figure 2).

In studies in patients with CVD (Stanek et al., 2011; Moore et al., 2015) simultaneous increase in brain perfusion and cognitive functions were observed. Moreover, also in CKD (Stringuetta Belik et al., 2018) which is typically associated with CVD, both perfusion and cognition improved. The main limitation of these studies is lack of direct assessment of causality between perfusion and cognition.

Most of the analyzed research agree with the fact that physical exercise has positive effect on general cognitive functioning as well as several specific cognitive domains such as executive functions and psychomotor speed. Those observations can be found especially in elderly subjects and patients with CVD, diabetes or chronic kidney diseased who are treated with haemodialysis. In patients with chronic kidney disease and stroke survivors after physical activity even improvement in global cognitive functioning can be seen. When taking in consideration that both renal dysfunction (Marini et al., 2021) and diabetes (Sigurdsson et al., 2021) are robustly associated with acute and chronic forms of cerebrovascular disease those results indicate that exercise may be helpful intervention especially in patients with high risk of cerebrovascular accidents.

While in patients with CVD or chronic somatic disorders cognitive decline might be visible and also cognitive improvement might be found even in general tests like MMSE or MoCA in healthy elderly subjects more sensitive test should be used. Majority of conducted studies included only singe test or various test measuring one function. Possibly wider range of tests might bring new insight in understanding impact of physical exercise and CBF on cognition. Especially memory functions might be important aspect needing more research. From all included studies only four examined learning and memory but only two found significant improvement after physical exercises (Guadagni et al., 2020; Lehmann et al., 2020).

CBF in healthy individuals does not show much connection with cognition. This is not surprising as in healthy subjects, there is a relative oversupply in terms of blood flow through the brain (Hall et al., 2016). Improvements in information processing speed and caution were reported after acute exposure to exercise.

Physical exercise affects the brain on anatomic, cellular and molecular levels which can enhance learning, memory and brain plasticity (De la Rosa et al., 2020). The potential protective mechanisms of exercise on brain aging are lowering levels of oxidative stress, regulating hormonal response, stimulating neurogenesis, elevating levels of neurotrophic factors and increasing CBF (Van Praag et al., 1999; Cotman and Berchtold, 2002; Colcombe and Kramer, 2003; Farmer et al., 2004; Heyn et al., 2004; Weuve et al., 2004; Eggermont et al., 2006; Deslandes et al., 2009). The association between exercise, cognitive functions and some of these factors have already been reviewed.

BDNF production and secretion are increased as a result of physical activity, and more importantly, BDNF has a causative role in the cognitive improvement induced by exercise. Furthermore, low serum levels of BDNF in humans have been linked to neurodegenerative diseases, such as AD, and high levels of BDNF are associated with increased hippocampal volume (Walsh and Tschakovsky, 2018; Tari et al., 2019).

Lactate is an important energy substrate in astrocytes, and at the same time, it plays a protective or modulatory role at the level of primary cortical areas (such as M1, V1, or S1) (Coco et al., 2020). Regular physical exercise diminishes blood-brain barrier permeability as it reinforces anti-oxidative capacity, reduces oxidative stress and has anti-inflammatory effects resulting in enhanced cognitive functions (Małkiewicz et al., 2019).

CBF changes are observed during acute physical exercise and following a long-term exercise program. Global CBF during exercise is primarily regulated by momentary arterial carbon dioxide partial pressure and the interactions of blood pressure and neurogenic activity (Smith and Ainslie, 2017). The effects of regular physical exercise are constantly investigated for its effects on brain function, which might be linked with improved cerebrovascular plasticity (Nishijima et al., 2016), maximal oxygen uptake (VO_2_max), cardiac output and arterial pressure (Querido and Sheel, 2007).

## Conclusions and Future Directions

Elevating CBF may induce improvement in conditions in which hypoperfusion is potentially a limiting factor; for instance, in patients suffering from cardiovascular abnormalities. Defining clinical conditions in which CBF decline is a limiting factor of cognitive impairment might be particularly clinically important because it could assist in the design of effective preventive and therapeutic programmes for patients.

Most of the studies found positive effect of physical activity on cognitive functioning, especially in executive functions and psychomotor speed, in elderly subjects and patients with chronic disorders with higher risk of cerebrovascular disease. Those results support hypothesis that exercise may bring eligible therapeutic effect in patients with high risk of cerebrovascular accidents.

The available data demonstrate that the relationship between exercise-induced improvements in perfusion and cognition has yet to be studied. In none of the reviewed studies was such association the primary endpoint of the study.

Carefully designed clinical studies focusing on cognitive and perfusion variables are needed to provide a response to the question whether exercise-induced cerebral perfusion augmentation is of clinical importance. Wider range of neuropsychological tests, especially assessing memory functions might bring important insight in understanding impact of physical exercise and cerebral blood flow on cognition. Such studies are urgently needed as physical exercise is widely recognized as relatively low-cost and safe supporting therapy in many cardiovascular and neurodegenerative disorders.

## Data Availability Statement

The original contributions presented in the study are included in the article/supplementary material, further inquiries can be directed to the corresponding author/s.

## Author Contributions

AM and PW: conceptualization. MR: methodology, investigation, writing—original draft preparation, and project administration. AM, SK, and PW: validation and supervision. MR and AM: formal analysis. PW: resources. MR, SK, and PW: funding acquisition. All authors: writing—review and editing, read, and agreed to the published version of the manuscript.

## Funding

MR is supported by the National Research and Development Centre (NCBiR) program, grant number POWR.03.05.00-00-z082/18. SK and AM are supported by the Polish National Science Centre (NCN) grant OPUS, number 2019/33/B/NZ7/01980.

## Conflict of Interest

The authors declare that the research was conducted in the absence of any commercial or financial relationships that could be construed as a potential conflict of interest.

## Publisher's Note

All claims expressed in this article are solely those of the authors and do not necessarily represent those of their affiliated organizations, or those of the publisher, the editors and the reviewers. Any product that may be evaluated in this article, or claim that may be made by its manufacturer, is not guaranteed or endorsed by the publisher.

## References

[B1] Abbenhardt C. Mctiernan A. Alfano C. M. Wener M. H. Campbell K. L. Duggan C. . (2013). Effects of individual and combined dietary weight loss and exercise interventions in postmenopausal women on adiponectin and leptin levels. J. Intern. Med. 274, 163–175. 10.1111/joim.12062 23432360 PMC3738194

[B2] Ainslie P. N. Cotter J. D. George K. P. Lucas S. Murrell C. Shave R. . (2008). Elevation in cerebral blood flow velocity with aerobic fitness throughout healthy human ageing. J. Physiol. 586, 4005–4010. 10.1113/jphysiol.2008.158279 18635643 PMC2538930

[B3] Alexander G. E. Furey M. L. Grady C. L. Pietrini P. Brady D. R. Mentis M. J. . (1997). Association of premorbid intellectual function with cerebral metabolism in Alzheimer's disease: implications for the cognitive reserve hypothesis. Am. J. Psychiatry. 154, 165–172. 10.1176/ajp.154.2.165 9016263

[B4] Alzheimer's Association (2018). 2018 Alzheimer's disease facts and figures special report: financial and personal benefits of early diagnosis. Alzheimers Dement 14, 367–429. 10.1016/j.jalz.2018.02.001

[B5] Alzheimer's Association (2019). 2019 Alzheimer's disease facts and figures. Alzheimer's Dement 15, 321–387. 10.1016/j.jalz.2019.01.010 22404854

[B6] Asllani I. Habeck C. Scarmeas N. Borogovac A. Brown T. R. Stern Y. (2008). Multivariate and univariate analysis of continuous arterial spin labeling perfusion MRI in Alzheimer's disease. J. Cereb. Blood Flow. Metab. 28, 725–736. 10.1038/sj.jcbfm.9600570 17960142 PMC2711077

[B7] Austin B. P. Nair V. A. Meier T. B. Xu G. Rowley H. A. Carlsson C. M. . (2011). Effects of hypoperfusion in Alzheimers Disease. J. Alzheimers Dis. 26, 123–133. 10.3233/JAD-2011-0010 21971457 PMC3303148

[B8] Barnes J. N. (2015). Exercise, cognitive function, and aging. Adv. Physiol. Educ. 39, 55–62. 10.1152/advan.00101.2014 26031719 PMC4587595

[B9] Baron J. C. Lebrun P. Collard P. Crouzel C. Mestelan G. (1982). Noninvasive Measurement of BloodFlow, Oxygen Consumption, andGlucose Utilization in the same brain regions in man by positron emission tomography: concise Communication. 23, 391–400. 6978932

[B10] Berent S. Giordani B. Lehtinen S. Markel D. Penney J. B. Buchtel H. A. . (1988). Positron emission tomographic scan investigations of Huntington's disease: cerebral metabolic correlates of cognitive function. Ann. Neurol. 23, 541–546. 10.1002/ana.410230603 2970247

[B11] Bertsch K. Hagemann D. Hermes M. Walter C. Khan R. Naumann E. (2009). Resting cerebral blood flow, attention, and aging. Brain Res. 1267, 77–88. 10.1016/j.brainres.2009.02.053 19272361

[B12] Binnewijzend M. A. A. Kuijer J. P. A. Benedictus M. R. Van Der Flier W. M. Wink A. M. Wattjes M. P. . (2013). Cerebral blood flow measured with 3D pseudocontinuous arterial spin-labeling MR imaging in alzheimer disease and mild cognitive impairment: a marker for disease severity. Radiology 267, 221–230. 10.1148/radiol.12120928 23238159

[B13] Burdette J. H. Laurienti P. J. Espeland M. A. Morgan A. Telesford Q. Vechlekar C. D. . (2010). Using network science to evaluate exercise-associated brain changes in older adults. Front. Aging Neurosci. 2, 23. 10.3389/fnagi.2010.00023 20589103 PMC2893375

[B14] Busch H. J. Schirmer S. H. Jost M. Van Stijn S. Peters S. L. M. Piek J. J. . (2011). Leptin augments cerebral hemodynamic reserve after three-vessel occlusion: distinct effects on cerebrovascular tone and proliferation in a nonlethal model of hypoperfused rat brain. J. Cereb. Blood Flow. Metab. 31, 1085–1092. 10.1038/jcbfm.2010.192 20978518 PMC3070967

[B15] Caplan L. R. Hennerici M. (1998). Impaired clearance of emboli (washout) is an important link between hypoperfusion, embolism, and ischemic stroke. Arch. Neurol. 55, 1475–1482. 10.1001/archneur.55.11.1475 9823834

[B16] Castan-Laurell I. Dray C. Attané C. Duparc T. Knauf C. Valet P. (2011). Apelin, diabetes, and obesity. Endocrine 40, 1–9. 10.1007/s12020-011-9507-9 21725702

[B17] Castan-Laurell I. Vítkova M. Daviaud D. Dray C. Kováčiková M. Kovacova Z. . (2008). Effect of hypocaloric diet-induced weight loss in obese women on plasma apelin and adipose tissue expression of apelin and APJ. Eur. J. Endocrinol. 158, 605–610. 10.1530/EJE-08-0039 18390990 PMC2683032

[B18] Castellano C. A. Paquet N. DIonne I. J. Imbeault H. Langlois F. Croteau E. . (2017). A 3-month aerobic training program improves brain energy metabolism in mild Alzheimer's disease: preliminary results from a neuroimaging study. J. Alzheimers Dis. 56, 1459–1468. 10.3233/JAD-161163 28157102

[B19] Chang Y. K. Labban J. D. Gapin J. I. Etnier J. L. (2012). The effects of acute exercise on cognitive performance: a meta-analysis. Brain Res. 1453, 87–101. 10.1016/j.brainres.2012.02.068 22480735

[B20] Cho S. Y. Roh H. T. (2019). Taekwondo enhances cognitive function as a result of increased neurotrophic growth factors in elderly women. Int. J. Environ. Res. Public Health. 16, 962. 10.3390/ijerph16060962 30889827 PMC6466246

[B21] Cho S. Y. So W. Y. Roh H. T. (2017). The effects of taekwondo training on peripheral Neuroplasticity-Related growth factors, cerebral blood flow velocity, and cognitive functions in healthy children: a randomized controlled trial. Int. J. Environ. Res. Public Health 14, 1–10. 10.3390/ijerph14050454 28441325 PMC5451905

[B22] Coco M. Buscemi A. Ramaci T. Tusak M. Di Corrado D. Perciavalle V. . (2020). Influences of blood lactate levels on cognitive domains and physical health during a sports stress. Brief review. Int. J. Environ. Res. Public Health 17, 9043. 10.3390/ijerph17239043 33291577 PMC7729439

[B23] Colcombe S. Kramer A. F. (2003). Fitness effects on the cognitive function of older adults: a meta-analytic study. Psychol. Sci. 14, 125–130. 10.1111/1467-9280.t01-1-01430 12661673

[B24] Cotman C. W. Berchtold N. C. (2002). Exercise: a behavioral intervention to enhance brain health and plasticity. Trends Neurosci. 25, 295–301. 10.1016/S0166-2236(02)02143-4 12086747

[B25] Creager M. A. Lüscher T. F. Cosentino F. Beckman J. A. (2003). Diabetes and vascular disease. Pathophysiology, clinical consequences, and medical therapy: Part I. Circulation 108, 1527–1532. 10.1007/978-1-4757-4564-1 14504252

[B26] Dai W. Lopez O. L. Carmichael O. T. Becker J. T. Kuller L. H. Gach H. M. (2008). Abnormal regional cerebral blood flow in cognitively normal elderly subjects with hypertension. Stroke 39, 349–354. 10.1161/STROKEAHA.107.495457 18174483 PMC2701215

[B27] Daulatzai M. A. (2017). Cerebral hypoperfusion and glucose hypometabolism: key pathophysiological modulators promote neurodegeneration, cognitive impairment, and Alzheimer's disease. J. Neurosci. Res. 95:943–972. 10.1002/jnr.23777 27350397

[B28] Daviglus M. L. Bell C. C. Berrettini W. Bowen P. E. Connolly E. S. Cox N. J. . (2010). NIH state-of-the-science conference statement: preventing Alzheimer's disease and cognitive decline. Ann. Intern. Med. 153, 176–181. 10.7326/0003-4819-153-3-201008030-00260 20547888

[B29] Davis C. L. Tomporowski P. D. McDowell J. E. Austin B. P. Miller P. H. Yanasak N. E. . (2011). Exercise improves executive function and achievement and alters brain activation in overweight children: a randomized, controlled trial. Heal. Psychol. 30, 91–98. 10.1037/a0021766 21299297 PMC3057917

[B30] De la Rosa A. Olaso-Gonzalez G. Arc-Chagnaud C. Millan F. Salvador-Pascual A. García-Lucerga C. . (2020). Physical exercise in the prevention and treatment of Alzheimer's disease. J. Sport Heal. Sci. 9, 394–404. 10.1016/j.jshs.2020.01.004 32780691 PMC7498620

[B31] Decroix L. Tonoli C. Soares D. D. Tagougui S. Heyman E. Meeusen R. (2016). Acute cocoa flavanol improves cerebral oxygenation without enhancing executive function at rest or after exercise. Appl. Physiol. Nutr. Metab. 41, 1225–1232. 10.1139/apnm-2016-0245 27849355

[B32] Deslandes A. Moraes H. Ferreira C. Veiga H. Silveira H. Mouta R. . (2009). Exercise and mental health: many reasons to move. Neuropsychobiology 59, 191–198. 10.1159/000223730 19521110

[B33] Dos Santos Picanco L. C. Ozela P. F. de Fatima de Brito Brito M. Pinheiro A. A. Padilha E. C. Braga F. S. . (2016). Alzheimer's disease: a review from the pathophysiology to diagnosis, new perspectives for pharmacological treatment. Curr. Med. Chem. 25, 3141–3159. 10.2174/0929867323666161213101126 30191777

[B34] Du A. T. Jahng G. H. Hayasaka S. Kramer J. H. Rosen H. J. Gorno-Tempini M. L. . (2006). Hypoperfusion in frontotemporal dementia and Alzheimer disease by arterial spin labeling MRI. Neurology 67, 1215–1220. 10.1212/01.wnl.0000238163.71349.78 17030755 PMC1779761

[B35] Eggermont L. Swaab D. Luiten P. Scherder E. (2006). Exercise, cognition and Alzheimer's disease: more is not necessarily better. Neurosci. Biobehav. Rev. 30, 562–575. 10.1016/j.neubiorev.2005.10.004 16359729

[B36] Ehlers D. Trinh L. McAuley E. (2016). The intersection of cancer and aging: implications for physical activity and cardiorespiratory fitness effects on cognition. Expert Rev. Qual. Life Cancer Care. 1, 347–350. 10.1080/23809000.2016.1241661

[B37] Espeland M. A. Luchsinger J. A. Neiberg R. H. Carmichael O. Laurienti P. J. Pi-Sunyer X. . (2018). Long term effect of intensive lifestyle intervention on cerebral blood flow. J. Am. Geriatr. Soc. 66, 120–126. 10.1111/jgs.15159 29082505 PMC5777883

[B38] Etgen T. Chonchol M. Frstl H. Sander D. (2012). Chronic kidney disease and cognitive impairment: a systematic review and meta-analysis. Am. J. Nephrol. 35, 474–482. 10.1159/000338135 22555151

[B39] Farmer J. Zhao X. Van Praag H. Wodtke K. Gage F. H. Christie B. R. (2004). Effects of voluntary exercise on synaptic plasticity and gene expression in the dentate gyrus of adult male sprague-dawley rats *in vivo*. Neuroscience 124, 71–79. 10.1016/j.neuroscience.2003.09.029 14960340

[B40] Ferris S. H. de Leon M. J. Wolf A. P. Farkas T. Christman D. R. Reisberg B. . (1980). Positron emission tomography in the study of aging and senile dementia. Neurobiol. Aging 1, 127–131. 10.1016/0197-4580(80)90005-6 24279935

[B41] Gligoroska J. Manchevska S. (2012). The effect of physical activity on cognition - physiological mechanisms. Mater Socio Medica 24, 198–202. 10.5455/msm.2012.24.198-202 23678325 PMC3633396

[B42] Guadagni V. Drogos L. L. Tyndall A. V. Davenport M. H. Anderson T. J. Eskes G. A. . (2020). Aerobic exercise improves cognition and cerebrovascular regulation in older adults. Neurology 94, e2245–e2257. 10.1212/WNL.0000000000009478 32404355 PMC7357295

[B43] Guan Z. Zhang M. Zhang Y. Li B. Li Y. (2021). Distinct functional and metabolic alterations of DMN subsystems in Alzheimer's disease: a simultaneous FDG-PET/fMRI study. 43rd Annu Int Conf IEEE Eng Med Biol Soc 2021, 3443–3446. 10.1109/EMBC46164.2021.9629472 34891980

[B44] Gunstad J. MacGregor K. L. Paul R. H. Poppas A. Jefferson A. L. Todaro J. F. . (2005). Cardiac rehabilitation improves cognitive performance in older adults with cardiovascular disease. J. Cardiopulm. Rehabil. 25, 173–176. 10.1097/00008483-200505000-00009 15931022 PMC3226758

[B45] Hall C. N. Howarth C. Kurth-Nelson Z. Mishra A. (2016). Interpreting BOLD: Towards a dialogue between cognitive and cellular neuroscience. Philos. Trans. R. Soc. B Biol. Sci. 371, 20150348. 10.1098/rstb.2015.0348 27574302 PMC5003850

[B46] Hebert L. E. Bienias J. L. Aggarwal N. T. Wilson R. S. Bennett D. A. Shah R. C. . (2010). Change in risk of Alzheimer disease over time. Neurology 75, 786–791. 10.1212/WNL.0b013e3181f0754f 20805524 PMC2938969

[B47] Hebert L. E. Weuve J. Scherr P. A. Evans D. A. (2013). Alzheimer disease in the United States (2010-2050) estimated using the 2010 census. Neurology 80, 1778–1783. 10.1212/WNL.0b013e31828726f,5 23390181 PMC3719424

[B48] Heo S. Prakash R. S. Voss M. W. Erickson K. I. Ouyang C. Sutton B. P. . (2010). Resting hippocampal blood flow, spatial memory and aging. Brain Res. 1315, 119–127. 10.1016/j.brainres.2009.12.020 20026320 PMC2822086

[B49] Heyn P. Abreu B. C. Ottenbacher K. J. (2004). The effects of exercise training on elderly persons with cognitive impairment and dementia: a meta-analysis. Arch. Phys. Med. Rehabil. 85, 1694–1704. 10.1016/j.apmr.2004.03.019 15468033

[B50] Hutchison J. L. Lu H. Rypma B. (2013). Neural mechanisms of age-related slowing: the ΔCBF/ΔCMRO 2 ratio mediates age-differences in BOLD signal and human performance. Cereb. Cortex 23, 2337–2346. 10.1093/cercor/bhs233 22879349 PMC3767961

[B51] Janelsins M. C. Kesler S. R. Ahles T. A. Morrow G. R. (2014). Prevalence, mechanisms, and management of cancer-related cognitive impairment. Int. Rev. Psychiatry 26, 102–113. 10.3109/09540261.2013.864260 24716504 PMC4084673

[B52] Jefferson A. L. Poppas A. Paul R. H. Cohen R. A. (2007). Systemic hypoperfusion is associated with executive dysfunction in geriatric cardiac patients. Neurobiol. Aging 28, 477–483. 10.1016/j.neurobiolaging.2006.01.001 16469418 PMC2741683

[B53] Johnson N. A. Jahng G. H. Weiner M. W. Miller B. L. Chui H. C. Jagust W. J. . (2005). Pattern of cerebral hypoperfusion in Alzheimer disease and mild cognitive impairment measured with arterial spin-labeling MR imaging: initial experience. Radiology 234, 851–859. 10.1148/radiol.2343040197 15734937 PMC1851934

[B54] Kalaria R. N. (1996). Cerebral vessels in ageing and Alzheimer's disease. Pharmacol. Ther. 72, 193–214. 10.1016/S0163-7258(96)00116-7 9364575

[B55] Kernan W. N. Ovbiagele B. Black H. R. Bravata D. M. Chimowitz M. I. Ezekowitz M. D. . (2014). Guidelines for the prevention of stroke in patients with stroke and transient ischemic attack: a guideline for healthcare professionals from the American Heart Association/American Stroke Association. Stroke 45, 2160–2236. 10.1161/STR.0000000000000024 24788967

[B56] Khazaei M. Tahergorabi Z. (2015). Leptin and its cardiovascular effects: focus on angiogenesis. Adv. Biomed. Res. 4, 79. 10.4103/2277-9175.156526 26015905 PMC4434486

[B57] Kisler K. Nelson A. R. Montagne A. Zlokovic B. V. (2017). Cerebral blood flow regulation and neurovascular dysfunction in Alzheimer disease. Nat. Rev. Neurosci. 18, 419–434. 10.1038/nrn.2017.48 28515434 PMC5759779

[B58] Kurella Tamura M. Wadley V. Yaffe K. McClure L. A. Howard G. Go R. . (2008). Kidney function and cognitive impairment in US adults: the reasons for geographic and racial differences in stroke (REGARDS) study. Am. J. Kidney. Dis. 52, 227–234. 10.1053/j.ajkd.2008.05.004 18585836 PMC2593146

[B59] Kurella Tamura M. Xie D. Yaffe K. Cohen D. L. Teal V. Kasner S. E. . (2011). Vascular risk factors and cognitive impairment in chronic kidney disease: the chronic renal insufficiency cohort (CRIC) study. Clin. J. Am. Soc. Nephrol. 6, 248–256. 10.2215/CJN.02660310 20930087 PMC3052213

[B60] Lacalle-Aurioles M. Mateos-Pérez J. M. Guzmán-De-Villoria J. A. Olazarán J. Cruz-Orduña I. Alemán-Gómez Y. . (2014). Cerebral blood flow is an earlier indicator of perfusion abnormalities than cerebral blood volume in Alzheimer's disease. J. Cereb. Blood Flow Metab. 34, 654–659. 10.1038/jcbfm.2013.241 24424381 PMC3982085

[B61] Leenders K. L. Perani D. Lammertsma A. A. Heather J. D. Buckingham P. Jones T. . (1990). Cerebral blood flow, blood volume and oxygen utilization: normal values and effect of age. Brain 113, 27–47. 10.1093/brain/113.1.27 2302536

[B62] Lefferts W. K. Babcock M. C. Tiss M. J. Ives S. J. White C. N. Brutsaert T. D. . (2016). Effect of hypoxia on cerebrovascular and cognitive function during moderate intensity exercise. Physiol. Behav. 165, 108–118. 10.1016/j.physbeh.2016.07.003 27402021

[B63] Lehmann N. Villringer A. Taubert M. (2020). Colocalized white matter plasticity and increased cerebral blood flow mediate the beneficial effect of cardiovascular exercise on long-term motor learning. J. Neurosci. 40, 2416–2429. 10.1523/JNEUROSCI.2310-19.2020 32041897 PMC7083530

[B64] Librizzi D. Cabanel N. Zavorotnyy M. Riehl E. Kircher T. Luster M. . (2021). Clinical relevance of [18F]Florbetaben and [18F]FDG PET/CT imaging on the management of patients with dementia. Molecules 26, 1–10. 10.3390/molecules26051282 33652938 PMC7956266

[B65] Lingzhong M. Wugang H. Jason C. Ruquan H. Adrian W. G. (2015). Cardiac output and cerebral blood flow: the integrated regulation of brain perfusion in adult humans. Anesthesiology 123, 1198–1208. 26402848 10.1097/ALN.0000000000000872

[B66] Loane D. J. Kumar A. (2016). Microglia in the TBI brain: the good, the bad, and the dysregulated. Exp. Neurol. 275, 316–327. 10.1016/j.expneurol.2015.08.018 26342753 PMC4689601

[B67] Lu B. Nagappan G. Lu Y. (2015). BDNF and synaptic plasticity, cognitive function, and dysfunction. Handb. Exp. Pharmacol. 220, 223–250. 10.1007/978-3-642-45106-5_9 24668475

[B68] Małkiewicz M. A. Szarmach A. Sabisz A. Cubała W. J. Szurowska E. Winklewski P. J. (2019). Blood-brain barrier permeability and physical exercise. J. Neuroinflam. 16, 15. 10.1186/s12974-019-1403-x 30678702 PMC6345022

[B69] Marini S. Georgakis M. K. Anderson C. D. (2021). Interactions between kidney function and cerebrovascular disease: vessel pathology that fires together wires together. Front. Neurol. 12, 1–13. 10.3389/fneur.2021.785273 34899586 PMC8652045

[B70] Mcmorris T. Hale B. J. (2012). Differential effects of differing intensities of acute exercise on speed and accuracy of cognition: a meta-analytical investigation. Brain Cogn. 80, 338–351. 10.1016/j.bandc.2012.09.001 23064033

[B71] Moore S. A. Hallsworth K. Jakovljevic D. G. Blamire A. M. He J. Ford G. A. . (2015). Effects of community exercise therapy on metabolic, brain, physical, and cognitive function following stroke: a randomized controlled pilot trial. Neurorehabil. Neural. Repair. 29, 623–635. 10.1177/1545968314562116 25538152

[B72] Moser D. J. Cohen R. A. Clark M. M. Aloia M. S. Tate B. A. Stefanik S. . (1999). Neuropsychological functioning among cardiac rehabilitation patients. J. Cardiopulm. Rehabil. 19, 91–97. 10.1097/00008483-199903000-00002 10200914

[B73] Moser D. J. Hoth K. F. Robinson R. G. Paulsen J. S. Sinkey C. A. Benjamin M. L. . (2004). Blood vessel function and cognition in elderly patients with atherosclerosis. Stroke 35, e369–e372. 10.1161/01.STR.0000145050.35039.51 15472091

[B74] Murray A. M. Tupper D. E. Knopman D. S. Gilbertson D. T. Pederson S. L. Li S. . (2006). Cognitive impairment in hemodialysis patients is common. Neurology 67, 216–223. 10.1212/01.wnl.0000225182.15532.40 16864811

[B75] Murrell C. J. Cotter J. D. Thomas K. N. Lucas S. J. E. Williams M. J. A. Ainslie P. N. (2013). Cerebral blood flow and cerebrovascular reactivity at rest and during sub-maximal exercise: Effect of age and 12-week exercise training. Age (Omaha) 35, 905–920. 10.1007/s11357-012-9414-x 22669592 PMC3636405

[B76] Nealon R. S. Howe P. R. C. Jansen L. Garg M. Wong R. H. X. (2017). Impaired cerebrovascular responsiveness and cognitive performance in adults with type 2 diabetes. J. Diab. Compl. 31:462–467. 10.1016/j.jdiacomp.2016.06.025 27431891

[B77] Nishijima T. Torres-Aleman I. Soya H. (2016). Exercise and cerebrovascular plasticity. Prog. Brain Res. 225, 243–268. 10.1016/bs.pbr.2016.03.010 27130419

[B78] Northey J. M. Pumpa K. L. Quinlan C. Ikin A. Toohey K. Smee D. J. . (2019). Cognition in breast cancer survivors: a pilot study of interval and continuous exercise. J. Sci. Med. Sport 22, 580–585. 10.1016/j.jsams.2018.11.026 30554923

[B79] Olivo G. Nilsson J. Garzón B. Lebedev A. Wåhlin A. Tarassova O. . (2021). Immediate effects of a single session of physical exercise on cognition and cerebral blood flow: a randomized controlled study of older adults. Neuroimage 225, 117500. 10.1016/j.neuroimage.2020.117500 33169699

[B80] Page M. J. McKenzie J. E. Bossuyt P. M. Boutron I. Hoffmann T. C. Mulrow C. D. . (2021). The PRISMA 2020 statement: an updated guideline for reporting systematic reviews. BMJ. (2021) 372, n71. 10.1136/bmj.n71 33782057 PMC8005924

[B81] Parkes L. M. Rashid W. Chard D. T. Tofts P. S. (2004). Normal cerebral perfusion measurements using arterial spin labeling: reproducibility, stability, and age and gender effects. Magn. Reson. Med. 51, 736–743. 10.1002/mrm.20023 15065246

[B82] Pasley B. N. Inglis B. A. Freeman R. D. (2007). Analysis of oxygen metabolism implies a neural origin for the negative BOLD response in human visual cortex. Neuroimage 36, 269–276. 10.1016/j.neuroimage.2006.09.015 17113313 PMC2001204

[B83] Pendergrass J. C. Targum S. D. Harrison J. E. (2018). Cognitive impairment associated with cancer: a brief review. Innov. Clin. Neurosci. 15, 36–44. 29497579 PMC5819720

[B84] Perry B. G. Lucas S. J. E. (2021). The acute cardiorespiratory and cerebrovascular response to resistance exercise. Sport Med. Open 7, 36. 10.1186/s40798-021-00314-w 34046740 PMC8160070

[B85] Querido J. S. Sheel A. W. (2007). Regulation of cerebral blood flow during exercise. Sport Med. 37, 765–782. 10.2165/00007256-200737090-00002 17722948

[B86] Rand D. Eng J. J. Tang P. F. Jeng J. S. Hung C. (2009). How active are people with stroke?: Use of accelerometers to assess physical activity. Stroke 40, 163–168. 10.1161/STROKEAHA.108.523621 18948606

[B87] Rosano C. Venkatraman V. K. Guralnik J. Newman A. B. Glynn N. W. Launer L. . (2010). Psychomotor speed and functional brain MRI 2 years after completing a physical activity treatment. J. Gerontol. A Biol. Sci. Med. Sci. 65, 639–647. 10.1093/gerona/glq038 20348185 PMC2869531

[B88] Ryan D. H. Espeland M. A. Foster G. D. Haffner S. M. Hubbard V. S. Johnson K. C. . (2003). Look AHEAD (Action for Health in Diabetes): design and methods for a clinical trial of weight loss for the prevention of cardiovascular disease in type 2 diabetes. Control Clin. Trials 24, 610–628. 10.1016/S0197-2456(03)00064-3 14500058

[B89] Sawicka M. Janowska J. Chudek J. (2016). Potential beneficial effect of some adipokines positively correlated with the adipose tissue content on the cardiovascular system. Int. J. Cardiol. 222, 581–589. 10.1016/j.ijcard.2016.07.054 27513655

[B90] Shimizu N. Umemura T. Matsunaga M. H. T. (2018). Effects of movement music therapy with a percussion instrument on physical and frontal lobe function in older adults with mild cognitive impairment: a randomized controlled trial. Aging Ment. Heal. 22, 1614–1626. 10.1080/13607863.2017.1379048 28937272

[B91] Sigurdsson S. Aspelund T. Kjartansson O. Gudmundsson E. Jonsson P. V. Buchem M. A. . (2021). Cerebrovascular risk-factors of prevalent and incident brain infarcts in the general population: the AGES-Reykjavik study. Stroke. 10.1161/STROKEAHA.121.034130. [Epub ahead of print]. 34809439 PMC8960318

[B92] Singh-Manoux A. Britton A. R. Marmot M. (2003). Vascular disease and cognitive function: evidence from the Whitehall II study. J. Am. Geriatr. Soc. 51, 1445–1450. 10.1046/j.1532-5415.2003.51464.x 14511166

[B93] Smith K. J. Ainslie P. N. (2017). Regulation of cerebral blood flow and metabolism during exercise. Exp. Physiol. 102, 1356–1371. 10.1113/EP086249 28786150

[B94] Stanek K. M. Gunstad J. Spitznagel M. B. Waechter D. Hughes J. W. Luyster F. . (2011). Improvements in cognitive function following cardiac rehabilitation for older adults with cardiovascular disease. Int. J. Neurosci. 121, 86–93. 10.3109/00207454.2010.531893 21062215

[B95] Stefanovic B. Warnking J. M. Pike G. B. (2004). Hemodynamic and metabolic responses to neuronal inhibition. Neuroimage 22, 771–778. 10.1016/j.neuroimage.2004.01.036 15193606

[B96] Steventon J. J. Hansen A. B. Whittaker J. R. Wildfong K. W. Nowak-Flück D. Tymko M. M. . (2018). Cerebrovascular function in the large arteries is maintained following moderate intensity exercise. Front. Physiol. 9, 1–9. 10.3389/fphys.2018.01657 30519192 PMC6258791

[B97] Stirling Meyer J. Rogers R. L. Judd B. W. Mortel K. F. Sims P. (1988). Cognition and cerebral blood flow fluctuate together in multi-infarct dementia. Stroke 19, 163–169. 10.1161/01.STR.19.2.163 3344529

[B98] Stringuetta Belik F. Oliveirae Silva V. R. Braga G. P. Bazan R. Perez Vogt B. Costa Teixeira Caramori J. . (2018). Influence of intradialytic aerobic training in cerebral blood flow and cognitive function in patients with chronic kidney disease: a pilot randomized controlled trial. Nephron 140, 9–17. 10.1159/000490005 29879707

[B99] Tari A. R. Norevik C. S. Scrimgeour N. R. Kobro-Flatmoen A. Storm-Mathisen J. Bergersen L. H. . (2019). Are the neuroprotective effects of exercise training systemically mediated? Prog. Cardiovasc. Dis. 62, 94–101. 10.1016/j.pcad.2019.02.003 30802460

[B100] Van Praag H. Christie B. R. Sejnowski T. J. Gage F. H. (1999). Running enhances neurogenesis, learning, and long-term potentiation in mice. Proc. Natl. Acad. Sci. USA. 96, 13427–13431. 10.1073/pnas.96.23.13427 10557337 PMC23964

[B101] Versari D. Daghini E. Virdis A. Ghiadoni L. Taddei S. (2009). Endothelial dysfunction as a target for prevention of cardiovascular disease. Diabetes Care 32, S314–S321. 10.2337/dc09-S330 19875572 PMC2811443

[B102] Walsh J. J. Tschakovsky M. E. (2018). Exercise and circulating BDNF: mechanisms of release and implications for the design of exercise interventions. Appl. Physiol. Nutr. Metab. 43, 1095–1104. 10.1139/apnm-2018-0192 29775542

[B103] Wang J. Sun H. Cui B. Yang H. Shan Y. Dong C. . (2021). The relationship among glucose metabolism, cerebral blood flow, and functional activity: a hybrid PET/fMRI study. Mol. Neurobiol. 58, 2862–2873. 10.1007/s12035-021-02305-0 33523358

[B104] Wefel J. S. Kesler S. R. Noll K. R. Schagen S. B. (2015). Clinical characteristics, pathophysiology, and management of noncentral nervous system cancer-related cognitive impairment in adults. CA Cancer J. Clin. 65, 123–138. 10.3322/caac.21258 25483452 PMC4355212

[B105] Weuve J. Kang J. H. Manson J. A. E. Breteler M. M. B. Ware J. H. Grodstein F. (2004). Physical activity, including walking, and cognitive function in older women. J. Am. Med. Assoc. 292, 1454–1461. 10.1001/jama.292.12.1454 15383516

[B106] WHO National Institute on Aging, National Institutes of Health, US, Department of Health and Human Services. (2011). Global Health and Ageing. (2011). Bethesda, MD: WHO, National Institute on Aging, National Institutes of Health, US Department of Health and Human Services.

[B107] Wolters F. J. Zonneveld H. I. Hofman A. Van Der Lugt A. Koudstaal P. J. Vernooij M. W. . (2017). Cerebral perfusion and the risk of dementia: a population-based study. Circulation 136, 719–728. 10.1161/CIRCULATIONAHA.117.027448 28588075

[B108] Yaffe K. Ackerson L. Tamura M. K. Le Blanc P. Kusek J. W. Sehgal A. R. . (2010). Chronic kidney disease and cognitive function in older adults: findings from the chronic renal insufficiency cohort cognitive study. J. Am. Geriatr. Soc. 58, 338–345. 10.1111/j.1532-5415.2009.02670.x 20374407 PMC2852884

[B109] Zhang N. Gordon M. L. Goldberg T. E. (2017). Cerebral blood flow measured by arterial spin labeling MRI at resting state in normal aging and Alzheimer's disease. Neurosci. Biobehav. Rev. 72, 168–175. 10.1016/j.neubiorev.2016.11.023 27908711

[B110] Zimmer P. Baumann F. T. Oberste M. Wright P. Garthe A. Schenk A. . (2016). Effects of exercise interventions and physical activity behavior on cancer related cognitive impairments: a systematic review. Biomed. Res. Int. 2016, 1820954. 10.1155/2016/1820954 27144158 PMC4842032

